# Attitudes of Healthcare Providers towards Non-initiation and Withdrawal of Neonatal Resuscitation for Preterm Infants in Mongolia

**DOI:** 10.3329/jhpn.v30i3.12298

**Published:** 2012-09

**Authors:** Ryan M. McAdams, Ariuntsatsral Erdenebileg, Maneesh Batra, Zagd Gerelmaa

**Affiliations:** ^1^University of Washington, Seattle, WA, USA; ^2^Shastin Central Hospital, Ulaanbaatar, Mongolia; ^3^Health Sciences University of Mongolia, Ulaanbataar, Mongolia

**Keywords:** Antenatal counselling, Ethics, Low-income country, Neonates, Resuscitation, Mongolia

## Abstract

Antenatal parental counselling by healthcare providers is recommended to inform parents and assist with decision-making before the birth of a child with anticipated poor prognosis. In the setting of a low-income country, like Mongolia, attitudes of healthcare providers towards resuscitation of high-risk newborns are unknown. The purpose of this study was to examine the attitudes of healthcare providers regarding ethical decisions pertaining to non-initiation and withdrawal of neonatal resuscitation in Mongolia. A questionnaire on attitudes towards decision-making for non-initiation and withdrawal of neonatal resuscitation was administered to 113 healthcare providers attending neonatal resuscitation training courses in 2009 in Ulaanbaatar, the capital and the largest city of Mongolia where ~40% of deliveries in the country occur. The questionnaire was developed in English and translated into Mongolian and included multiple choices and free-text responses. Participation was voluntary, and anonymity of the participants was strictly maintained. In total, 113 sets of questionnaire were completed by Mongolian healthcare providers, including neonatologists, paediatricians, neonatal and obstetrical nurses, and midwives, with 100% response rate. Ninety-six percent of respondents were women, with 73% of participants from Ulaanbaatar and 27% (all midwives) from the countryside. The majority (96%) of healthcare providers stated they attempt pre-delivery counselling to discuss potential poor outcomes when mothers present with preterm labour. However, most (90%) healthcare providers stated they feel uncomfortable discussing not initiating or withdrawing neonatal resuscitation for a baby born alive with little chance of survival. Religious beliefs and concerns about long-term pain for the baby were the most common reasons for not initiating neonatal resuscitation or withdrawing care for a baby born too premature or with congenital birth-defects. Most Mongolian healthcare providers provide antenatal counselling to parents regarding neonatal resuscitation. Additional research is needed to determine if the above-said difficulty with counselling stems from deficiencies in communication training and whether these same counselling-related issues exist in other countries. Future educational efforts in teaching neonatal resuscitation in Mongolia should incorporate culturally-sensitive training on antenatal counselling.

## INTRODUCTION

The American Academy of Pediatrics Committee on the Fetus and Newborn has recommended that each institution caring for women at risk of delivering extremely preterm infants should provide comprehensive and consistent guidelines for antenatal counselling (1). Antenatal counselling by healthcare providers is recommended to inform parents and assist with decision-making before the birth of a child with anticipated poor prognosis (1-7). Parents and care providers are often required to make difficult decisions about neonatal resuscitation (NR) when faced with a potential preterm delivery, especially since the infant's health outcome is not always predictable (3).

Care providers are often in position of power and authority and decide on controversial issues in medical, scientific or even legal matters (5), and not solely dependent on parental preferences. Care providers are influenced in their decisions by their social and professional backgrounds and also by their own fears and opinions regarding the value of life (8). Attitudes of neonatologists towards ethical issues surrounding decision-making for neonates at risk of death are influenced by practice in high-income countries. They are also influenced by similar practice in their own countries, religious beliefs, number of extremely-preterm infants they have cared for, and number of offspring of the concerned parents (9). Of the world's estimated 2 million preterm births each year, most occur in low-income countries (10), and it is not clear how attitudes of care providers towards neonatal viability influence antenatal counselling to parents regarding non-initiation or withdrawal of NR in these settings.

Attitudes towards antenatal counselling and the involvement of parents in collaborative decisions about NR may vary among individual care providers, different geographic settings, and between countries. Although antenatal parental counselling is recommended, not all care providers may be comfortable in performing this counselling, depending on their background and the sociocultural, political or legal settings (11). In Mongolia, consensus guidelines for antenatal counselling have not been established, and the role of care providers in making decisions about NR has not been previously studied. The purpose of this study was to evaluate the attitudes of care providers towards early decision-making on non-initiation and withdrawal of NR for preterm newborns. We discuss how these factors influence antenatal counselling and medical practice in Mongolia.

## MATERIALS AND METHODS

### Survey instrument

A questionnaire on attitudes and decision-making relating to NR was administered to 113 care providers in person. All care providers attending NR training courses or directly involved in NR at hospitals in Ulaanbaatar were eligible to participate in the questionnaire survey. The questionnaire was developed in English by one of the authors (Ryan McAdams) and translated into Mongolian by another author (Ariuntsatsral Erdenebileg) who is fluent in both languages. The questionnaire consisted of Likert Scale responses, yes/no, multiple choices, and open-ended questions. Participation was voluntary, and anonymity of the participants was strictly maintained. Results of the Likert Scale questions were combined to create dichotomous responses (high and low). For example, those responses coded 1 to 2 for “lack of proper medical training prevents resuscitation” were scored as high whereas those coded 4 to 5 were scored as low. Those that scored 3 were considered neither high nor low. Institutional review board of the University of Washington approved the questionnaire.

### Setting

Ulaanbaatar is the capital of Mongolia and home to 40% of the country's population. In 2007, approximately 31% (17,768/56,636) of deliveries in Mongolia occurred in Ulaanbaatar (data from United Nations Demographic Yearbook, United Nations Statistics Division, November 2009). At the time of the survey, there were 267 care providers in Ulaanbaatar involved in providing NR care. Care providers from Ulaanbaatar and those attending from the countryside were given the questionnaire at NR training courses held in 2009 at the First Maternity Hospital and the State Maternal and Child Health Research Center, two of the main hospitals in Mongolia for high-risk deliveries.

### Statistics

Proportions were compared with Fisher's exact test, using SPSS software (Chicago, IL, USA). All comparisons were two-tailed with alpha ≤0.05.

## RESULTS

In total, 113 (100% response rate) sets of questionnaire were completed by Mongolian care providers who were directly involved with making decisions on NR. The care providers included neonatologists, paediatricians, neonatal and obstetrical nurses, and midwives. Nearly all—96% (108/113)—of the respondents were women, with 73% (82/113) from Ulaanbaatar and 27% (31/113) from the countryside. All of them were midwives. The sets of questionnaire were completed by 31% (82/267) of care providers in Ulaanbaatar involved in providing NR care.

The majority of care providers (93%, 105/113) stated they attempt antenatal counselling to discuss potential poor outcomes when mothers present with preterm labour (Table 1). However, 90% (99/110) of the responding care providers stated they feel uncomfortable discussing initiation and stopping NR in a baby who is born alive but with little chance of survival (Table 2). The figure plots the earliest gestational age (GA) range at which Mongolian care providers reported they would provide NR. In total, 113 healthcare providers were queried, and 90 responded; one respondent who performed newborn resuscitation as early as 21 weeks of gestational age was not included in the analysis. As shown, 81% (72/89) of the respondents stated that they would perform NR at the gestational age of 30 weeks or earlier. The majority of care providers (68%, 75/110) responded that the plan for NR made during antenatal period is subject to change based on the initial assessment of the newborn. Most care providers (67%, 76/113) attempt to include family members in discussions during antenatal period, with 61% (68/112) stating that the mother has the most authority in making decisions. Almost all care providers (98%, 106/108) stated they were uncomfortable explaining reasons to the family why the baby died.

**Table. 1.** Percentage and number of ‘Yes’ and ‘No’ responses to key survey questions comparing urban and rural healthcare providers in Mongolia

Question	Responses: Yes % (Yes=n, No=n)		
	Urban	Rural	p value*
Do you attempt antenatal counselling?	94 (y=78, n=5)	100 (y=27, n=0)	0.331
Do you typically include any family members other than the mother in your prenatal counselling?	63 (y=50, n=30)	93 (y=26, n=2)	0.002
Do you attend all high-risk deliveries?	62 (y=50, n=31)	63 (y=17, n=10)	1.000
Does financial burden on the family play a role in the decision-making for NR?	32 (y=24, n=52)	8 (y=2, n=22)	0.030
After a newborn baby dies, do you explain to the family why you think the baby died?	97 (y=76, n=2)	90 (y=26, n=3)	0.122

*Fisher's exact test (two-tailed)

**Table. 2.** Comfort levels of Mongolian healthcare providers with parental counselling

Question	Response (n)					
How comfortable are you discussing NR…?	Completely	Very	Relatively	Not very	Not at all	Don't know
Do you discuss with parents about starting and stopping resuscitation in a baby that is born alive but has little chance of survival?	0.9% (1/110)	3.6% (4/110)	3.6% (4/110)	50.9% (56/110)	39.1% (43/110)	1.8% (2/110)
Do you discuss with parents about withdrawing life-support for a baby in the intensive care unit when death seems inevitable?	0%	3.6% (4/111)	6.3% (7/111)	45.9% (51/111)	36% (40/111)	8.1% (9/111)
Do you talk to parents of a newborn baby that died at birth?	0%	0%	0%	30.5% (33/108)	67.6% (73/108)	3.6% (4/111)

Religious beliefs (62%, 63/101) and concerns for long-term pain issues (34%, 34/101) were the most commonly-stated factors that influence care providers regarding non-initiation or withdrawal of NR when a newborn is premature or born with a birth-defect. Genetic defects, congenital heart disease, and mental impairment relating to prematurity were considered “the worst to have” in the Mongolian culture. Almost half of care providers (46% 49/106) felt that newborns with birth-defects are accepted like the ‘normal’ babies by society while 31% (33/106) disagreed, and 23% (24/106) had no opinion. Potential financial burden to the family does not influence most (74%, 74/100) decisions of care providers not to resuscitate a baby at high risk for long-term medical complications. Only 38% (33/88) of care providers felt there are good developmental service programmes available in Ulaanbaatar for children with disabilities. Most care providers agreed that lack of hospital resources (51%, 52/102) or medical training (78%, 81/104) does not prevent NR for high-risk babies in Ulaanbaatar.

**Fig.**
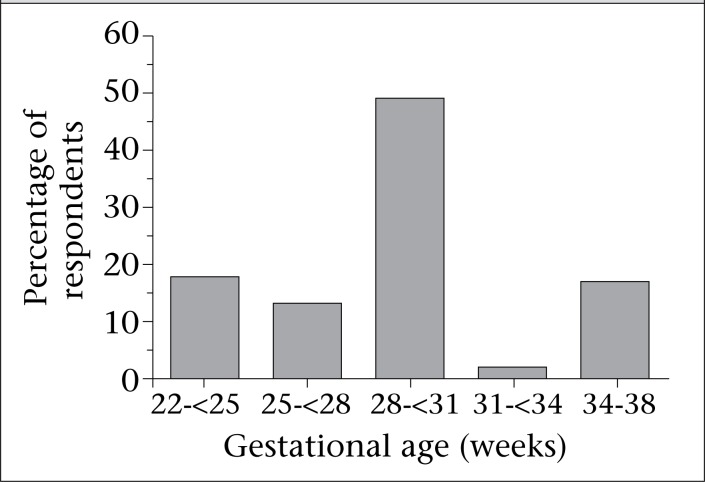
The earliest gestational age at which healthcare providers reported they would perform newborn resuscitation

Differences in aspects of antenatal counselling based on survey response were seen between care providers practising in Ulaanbaatar (urban healthcare providers) compared to those practising in the rural areas as demonstrated in Table 1. The majority of urban (94%) and rural (100%) care providers stated that they attempt antenatal counselling; however, rural care providers include other family members (in addition to the mother) in counselling more often than urban healthcare providers (93% versus 63%; p=0.002). Additionally, concerns about financial burden to families influence NR decision-making by urban healthcare providers (32%) more than the rural ones (8%, p=0.03). The average GA that care providers would consider to perform newborn resuscitation was not significantly different between urban and rural care providers (28.7 vs 27.3 weeks GA).

## DISCUSSION

We collected survey data to understand how antenatal counselling influences the decision-making process relating to NR in Mongolia. Our principal observation is that care providers in Mongolia routinely engage in discussions with parents to make antenatal decisions concerning adverse NR outcomes. These data are encouraging because the widespread use of antenatal counselling in low-income Mongolia would match the recommendations made in high-income countries (1-7) and also satisfy the desire of parents to be involved in decisions (12). Our survey also identified that most care providers in Mongolia feel uncomfortable discussing non-initiation or withdrawal of care for a baby born alive with little chance of survival as well as discussing with families why an infant died. Additionally, differences in aspects of antenatal counselling (such as including someone other than the mother in counselling) between care providers practising in Ulaanbaatar and in the countryside are reported in this study. Further research is needed to determine the differentials in what factors (e.g. socioeconomic, cultural and religious issues, or training) influence antenatal counselling between care providers practising in an urban setting, like Ulaanbaatar, and the countryside. Factors that impede antenatal counselling, including stress and communication, availability of guidelines, diagnostic resources, and morbidity data, are discussed below.

Counselling can be a stressful process for the care providers. Although most Mongolian care providers (96%) provide antenatal counselling to parents regarding NR, the majority of care providers (90%) feel uncomfortable discussing non-initiation and withdrawal of NR. Similarly, when a baby had died, the majority of providers (98%) stated they felt uncomfortable discussing death with family members. This feeling of discomfort when discussing death has not been well-studied in care providers involved in antenatal counselling. However, among professionals in mental health, situations involving death or loss are considered more distressing, triggering increased levels of personal distress compared to other counselling situations (13,14). Additional research may determine whether factors, such as communication training, viability criteria, ambiguity in determining gestational age, or knowledge of survival/mortality data, influence the degree of anxiety experienced by counsellors. Furthermore, since comfort with discussing non-initiation and withdrawal of NR and death does not imply competence, future studies aimed at assessing effective communication skills of healthcare providers faced with these situations will be of value.

Antenatal counselling guidelines must be flexible to account for various circumstances and available resources. Regardless of the setting, antenatal counselling on NR by healthcare providers should include the best estimate of the short-term and long-term benefits, contrasted with risks, and be based on relevant mortality and morbidity data. Decisions are more difficult when preterm labour is unexpected and when reliable and context specific survival and morbidity data are unavailable. Often, decisions regarding NR are made under stressful conditions; for example, when a pregnant woman presents with unexpected preterm labour. Decisions become more challenging when they require coordinated counselling between parents and medical team members (e.g. obstetricians, neonatologists, and midwives). These challenges exist in both high- and low-income countries. Given the need for region-specific data, universal counselling guidelines may not be possible.

The unavailability of prenatal diagnostic resources creates uncertainty that influences the ability to provide antenatal counselling. For example, prenatal ultrasound or *in vitro* fertilization are not routinely available in Mongolia. Therefore, GA estimates are less accurate since these are typically determined by maternal history and examination. Moreover, without diagnostic equipment, many factors remain unknown, including gender of the foetus, presence of oligo- or polyhydramnios, growth restriction, multiple gestation, and foetal anomalies. Correspondingly, this uncertainty is likely to affect antenatal counselling. Most of the care providers (68%, 75/110) we surveyed responded that the antenatal plan for NR is subject to change based on the initial assessment of the newborn. This modification of decisions by including a postnatal GA assessment and evaluating the newborn's response to resuscitation and stabilization seems appropriate, given the limited prenatal diagnostic capability. In some cases, NR may actually provide additional time to verify the estimated GA, improve prognostic confidence, and provide an additional opportunity for collective decision-making by the physicians and parents.

Many Mongolian care providers (49%, 44/89) stated that the earliest GA they would consider to attempt NR on a baby was between 28 and 30 weeks. However, 18% (16/89) said they would consider performing NR in babies down to 22-24 weeks of GA. ‘Viability thresholds’ are typically determined as a product of both foetal capacity to survive outside the womb, balanced by care provider and parental acceptance of necessary suffering to achieve ‘good’ outcomes. Both of these elements are context-specific and vary by country, region, and even from patient to patient. Therefore, establishment of strict ‘viability thresholds’ by hospitals and governing organizations has been appropriately elusive. In high-income countries, current viability thresholds are typically reported to be between 22 and 24 weeks of GA (15-17). The GA that is considered previable in Mongolia, as in many low-income countries, is not clear but is likely to vary within the country depending on factors, such as biomedical technological capacity, the expertise level of the local hospitals, available medical and financial resources, education of parents and care providers, and both cultural and religious beliefs. In low-income countries, it is not clear how knowledge of care providers about viability limits (22-23 weeks of gestation) compared to high-income countries affects decision-making regarding local viability limit thresholds or the willingness to attempt replication of published outcomes at the extremes of viability. Additionally, the influence of visiting care providers, NR training sessions, and donated medical equipment on decision-making pertaining to local viability limits is not known. Further research is needed to determine what factors influence changes in local attitudes towards viability limits in low-income countries.

In Mongolia, determining who is to be given resuscitation has significant implications for the health of the population, given the overall scarcity of resources for healthcare. Furthermore, a recent cross-sectional study on screening for disability in UNICEF's Multiple Indicator Cluster Survey programme reported that 26% of 4,921 Mongolian children aged 2-9 years screened were at risk of disability (18). Whether or not Mongolian care providers are aware of the prevalence of children who have neurodevelopmental disabilities is not known. However, only 38% (33/88) of Mongolian care providers felt there are good developmental services available in Ulaanbaatar. The degree to which the care providers perceive lack of resources available to children with disabilities and how much their families influence decision-making is also unknown.

Most Mongolian care providers agreed that lack of hospital resources (51%, 52/102) does not prevent NR for high-risk babies in Ulaanbaatar. However, it is unclear if limited resources affect care-giving decisions once a newborn is admitted to the Neonatal Intensive Care Unit. Interestingly, concerns of financial burden for the family had more of an influence on decision-making for NR by care providers practising in a city (32%) compared to the countryside (8%). Although more family resources may be available in a large city, like Ulaanbaatar, financial support systems (e.g. extended family or community support) may differ between city and countryside settings. However, further research is needed to determine how differences in urban versus rural settings affect antenatal counselling behaviours. The impact of factors such as finances and perceived futility on decision-making may be magnified in resource-poor regions and need to be better understood when considering care practices in different geographic and cultural settings.

### Limitations

A limitation of our study is that we did not collect demographic information, such as age, number of years in practice, religious preference, marital status, or number of children of care providers that may influence antenatal counselling attitudes. Additionally, not all care providers indicated their job titles, preventing subgroup analysis of responses by type of respondent. A study by Cummings *et al*. looking at opinions of neonatologists in Connecticut and Rhode Island about NR for extremely premature infants showed no significant relationships with gender, age, years in practice, being a parent, or religion (19). However, these data may not be indicative of the opinions of Mongolian care providers, especially since 62% (63/101) cited religious beliefs as the most common factor that influences decisions on non-initiation or withdrawal of NR. Although the questionnaire was only administered to care providers known to be directly involved in NR, only 62% of respondents stated that they attended high-risk deliveries. Additionally, although nearly 40% of all deliveries in Mongolia occur in Ulaanbaatar where 73% of the respondent care providers practise, the viewpoints in this study may not be generalizable to those in non-urban regions in Mongolia.

### Conclusions

There remains a paucity of information on many aspects of newborn's survival and standards of care in low-income countries, despite a growing global interest in these areas. A recent meta-analysis by Pignotti and Donzelli on perinatal care at the threshold of viability, comparing international practice guidelines for the treatment of extremely preterm births, reviewed 15 studies; none was from lower-income countries (15). This lack of data and guidelines for low-income countries reinforces the huge gap that exists in newborn's survival and standards of care in these countries compared to the high-income countries. Fortunately, NR training is increasing and being recognized as a missed opportunity for saving lives and for improving morbidity outcomes globally (20). Future efforts to establish international NR practice guidelines should address issues relating to care providers’ antenatal decision-making processes for non-initiation or withdrawal of NR. Furthermore, NR practice guidelines and estimates of mortality reduction relating to NR should extend beyond high-income countries, especially since the majority of infants in the world are born and die in the low-income countries.

### ACKNOWLEDGEMENTS

We would like to thank Ron McPherson for his help in preparing the manuscript.
